# The distinct cell physiology of *Bradyrhizobium* at the population and cellular level

**DOI:** 10.1186/s12866-024-03272-x

**Published:** 2024-04-20

**Authors:** Ian F. Medici, Leila Bartrolí, Francisco F. Guaimas, Fabiana R. Fulgenzi, Charo Luciana Molina, Ignacio Enrique Sánchez, Diego J. Comerci, Elías Mongiardini, Alfonso Soler-Bistué

**Affiliations:** 1 Instituto de Investigaciones Biotecnológicas, IIB-IIBIO, Universidad Nacional de San Martín- Consejo Nacional de Investigaciones Científicas y Técnicas (CONICET), Av. 25 de Mayo y Francia CP (1650), San Martín, Prov. de Buenos Aires, Argentina; 2 Laboratorio de Fisiología de Proteínas, Facultad de Ciencias Exactas y Naturales, CONICET Instituto de Química Biológica, Facultad de Ciencias Exactas y Naturales (IQUIBICEN), Universidad de Buenos Aires, Buenos Aires, Argentina; 3 Instituto de Biotecnología y Biología Molecular (IBBM), Facultad de Ciencias Exactas, UNLP y CCT-La Plata-CONICET, La Plata, Argentina

**Keywords:** *Bradyrhizobium*, Bacterial physiology, Growth rate, Cell polarity

## Abstract

The α-Proteobacteria belonging to *Bradyrhizobium* genus are microorganisms of extreme slow growth. Despite their extended use as inoculants in soybean production, their physiology remains poorly characterized. In this work, we produced quantitative data on four different isolates: *B. diazoefficens* USDA110, *B. diazoefficiens* USDA122, *B. japonicum* E109 and *B. japonicum* USDA6 which are representative of specific genomic profiles. Notably, we found conserved physiological traits conserved in all the studied isolates: (i) the lag and initial exponential growth phases display cell aggregation; (ii) the increase in specific nutrient concentration such as yeast extract and gluconate hinders growth; (iii) cell size does not correlate with culture age; and (iv) cell cycle presents polar growth. Meanwhile, fitness, cell size and in vitro growth widely vary across isolates correlating to ribosomal RNA operon number. In summary, this study provides novel empirical data that enriches the comprehension of the *Bradyrhizobium* (slow) growth dynamics and cell cycle.

**Supplementary Information:**

The online version contains supplementary material available at 10.1186/s12866-024-03272-x.

## Introduction

Duplication is the fundamental property of all living cells. Bacteria are the simplest cells and growth rate (GR) is the key parameter to model their duplication capacity, yield and stress tolerance. It reflects bacterial fitness, biochemistry and competitiveness [1]. The maximal GR varies widely across bacteria [2]. Fast-growers such as *Vibrio cholerae* display a generation time (GT, the elapsed time between successive divisions) of 17 min [3]. Meanwhile, slow-growing bacteria, such as *Bradyrhizobium*, can take weeks to develop in culture, making its study challenging and limiting their biotechnological utility. A recent study considers slow-growing microorganisms those having a GT > 5 h [4]. Despite more than a century studying bacterial physiology [5, 6], the genetic and genomic factors shaping GR remain an open question [4, 7]. Moreover, most of what we know of bacterial physiology comes from fast-growing organisms like *Escherichia coli, Salmonella* or *Bacillus* [5]. Another well-known bacterial system is *Caulobacter vibrioides* (formerly known as *C. crescentus*) which has been thoroughly characterized as model for α-Proteobacteria [8–10]. This latter group includes biotechnologically relevant microorganisms such as *Rhizobiaceae* since they are well-known as crop symbionts. Among rhizobia, *Bradyrhizobium* is a complex monophyletic clade of slow-growing bacteria [2, 11–14]. The genus is highly prevalent in soil and includes symbiotic and free-living microorganisms of great economic interest [15–18]. Its metabolism and its symbiotic interaction with crops, such as soybean or peanut, have been very well characterized but little is known about its growth physiology and cell cycle [19–21].

The bacterial cell physiology is addressed using the growth curve i.e., following optical density over time, as an estimator of bacterial abundance. Classically, four stages are recognized along bacterial growth curve since first characterized by Janet Lane-Claypon in 1909 [5, 6, 22]: the lag phase (I), the exponential growth phase (II), the stationary (III), and the decline phase (IV). Growth curve study allows determining key physiological parameters such as growth rate (or its inverse, the GT), the lag phase duration and, the maximum system carrying capacity. In the literature, *Bradyrhizobium* growth curves are typically performed by taking one OD measurement per day (e.g [23, 24]). While for general studies this sampling frequency provides enough information, detailed cell physiology studies require higher resolution. For instance, the doubling time, a key parameter of bacterial physiology, requires sampling at least twice per generation time, which in *Bradyrhizobium* ranges from 6 to 48 h. Additionally, since OD is an indirect method of estimating cell numbers, growth curves must be complemented with colony counting and/or microscopy studies.

In the present work, we chose four representative *Bradyrhizobium*, isolates from temperate land whose genomes are fully available: *B. diazoefficiens* USDA122 [25], *B. diazoefficiens* USDA110 [18, 26], *B. japonicum* USDA6 [27] and *B. japonicum* E109 [28]. We characterized the physiology of this isolates using several experimental approaches: manual and automatic growth curves, colony counting, microscopy, and pairwise competition experiments. *B. diazoefficiens* USDA110 (*Bd*110), *B. diazoefficiens* USDA122 (*Bd*122), *B. japonicum* USDA6 (*Bj*6) are the main representatives of specific genomic profiles regarding the presence of genomic islands [29]. Meanwhile, the *B. japonicum* E109 (*Bj*E109) has a similar profile to *Bj*U6 but it is highly used for soybean growth promotion in temperate regions [28]. Despite their high genome similarity, we observed some significant physiological differences among these isolates, which that we correlated to specific genomic features.

## Experimental procedures

### Growth conditions and quantification

the strains used in this study are summarized in Additional File 1: Table S1 in the supplements. *Bradyrhizobium* strains were grown aerobically in yeast extract-mannitol (YEM) [30], or the AG medium [30]. Total biomass was estimated by measurement of the optical density at 450 nm (OD_450nm_) and the number of viable bacteria by the number of CFU on YEM agar plates supplemented with Congo Red (10 mg/L) (YMA), using the microdilution method [31]. For manual growth curves, 50-mL cultures were grown in 250-mL Erlenmeyer flasks at 28 °C with rotary shaking at 200 rpm in contact with the air.

### Automated growth curve measurements

Saturated cultures of the indicated microorganism were diluted 1/1000 in culture media. Bacterial preparations were distributed in triplicate or quadruplicate in p96 microplates. Growth-curve experiments were performed using a Tecan Infinite Sunrise microplate reader at 28 °C, with absorbance measurements (450 nm) taken at 30-minute intervals for 5–7 days with agitation for 15 min. The slopes during exponential phase were directly obtained using GrowthRates program [32].

### Microscopy

Bacterial samples were placed on a PBS agar 1.5-2% m/v slide (or the described medium) and observed at the indicated growth phase. For cell size and fluorescence determinations, we employed a Nikon Eclipse T2000U light microscope at 640X to 1000X magnification.

For time-lapse microscopy, an aliquot was taken from an exponentially growing culture and placed in AG-agar medium slide sealed with VALAP resin (vaseline, linoleic acid, paraffin 1:1:1) [33]. A Zeiss Axiobserver 7 microscope, with an iPlan-Apochromat 63x oil M27 objective was automated every 30 min for 5 days. To maintain the focus along the experiment, a small region of interest (ROI) containing a cell was selected. Then, we programed the autofocus set up within the Zen Blue controller software (Zeiss) to find the plane that maximized the contrast. In case that contrast maximization failed, we registered 4 fields and 10 planes in Z axis using a 0.5 μm difference.

Confocal laser-scanning microscopy (CLSM), was performed in an Olympus FV1000 microscope employing the oil objective PlanApo N (60 × 1.42 NA). Images were taken in the XY plane along the Z axis using a z-step increment of 0.22 μm (Z-stack).

### Cell size measurement

To carry out the measurement, the strains *B. japonicum* E109 and *B. diazoefficiens* USDA110 were grown in YEM medium, starting from precultures and then cultivated with shaking at a temperature of 28 °C in a 50 mL Erlenmeyer flask. Aliquots were taken at different times and placed in PBS-agar medium on a slide. Images were taken on a Nikon Eclipse TE2000-U microscope. They were analyzed using FIJI [34].

### Competitive fitness assays

These assays were performed similarly as in [35] but microscopy instead of flow cytometry was utilized for determining the proportion of fluorescent cells in each sample. *B. japonicum* E109, *B. diazoeffeciens* USDA110, *B. japonicum* USDA6^T^ and *B. diazoefficiens* USDA122 were co-cultivated with a *Bd*110 expressing the green fluorescent (GFP^+^) by integration into its genome (Additional File 1: Table S1). For this, 3–4 days-old precultures were quantified by OD and mixed with *Bd*110 *gfp* + in a ∼ 1:1 proportion that was verified by fluorescent microscopy. The experiment started by using 15 µL of the mix to inoculate 15 mL of YEM medium in 50mL Erlenmeyer with each mix of cells composed of ∼ 50% of fluorescent *Bd*110 *gfp* + and ∼ 50% of each non-fluorescent strain tested. Subsequently, daily aliquots were taken, measuring the *gfp* + vs. *gfp*- ratio. *B. diazoeffeciens* USDA110 *gfp* + strain was grown alone to normalize the fluorescence value but practically no loss of fluorescence was observed. Fitness for each strain was calculated as follows $Wstrain=ln \left(\frac{Nfinal}{Ninitial}\right) /ln \left(\frac{Ngfp.final}{Ngfp.initial}\right)$ where W_strain_ is the fitness of the strain under study, *N*_initial_ and *N*_final_ are the quantity of the derivative strain before and after the competition (non-fluorescent) and *N*_*gfp*_._initial_ and *N*_*gfp*_._final_ are the numbers of cells of *B. diazoefficiens* USDA110 *gfp*+ before and after the competition. Final cell numbers were estimated using OD. The experiments were performed at least 3 times. Results were expressed as relative fitness (W_rel_) is the ratio of the W of each strain and the W of the reference strain, *B. diazoefficiens* USDA110 *gfp*+.

### Fluorescent D-amino acid (FDAA) labeling

The procedure was done as referred in [36]. *E. coli* was grown in LB at 37 °C. *A. fabrum C58* was grown in LB at 28 °C. Bradyrhizobia were cultured in AG at 28 °C. The saturated cultures were washed and a 1/1,000 dilution was made on the same medium. At exponential phase (OD ≈ 0.4) and a 750 µL aliquot was resuspended in 100 µL of fresh media and 5 µL of FDAA of a stock solution (5 µM) was added. As FDAA, green sulfonated BODIPY-FL 3-amino-D-alanine (sBADA) was used. This was incubated for 10% of GT. Cells are then washed twice and placed on a PBS agar 1.5-2% m/v slide to microscopy observation.

## Results

### Bradyrhizobia growth curve presents an abnormal lag phase

**Fig. 1:**
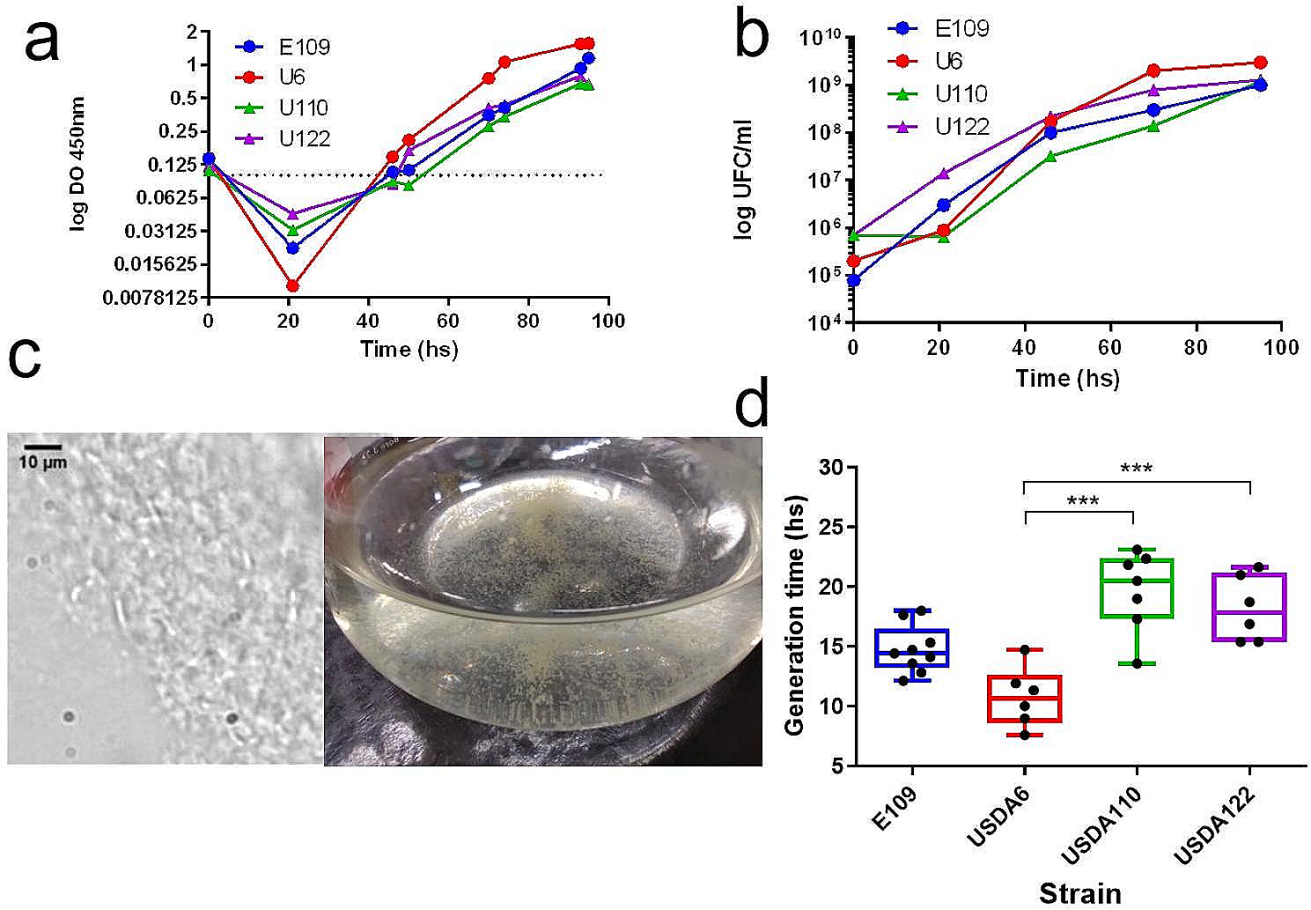
General features of*Bradyrhizobium* growth.**(a)** A representative growth curve manually performed by following OD_450nm_ along time in classical YEM medium of *B. japonicum* E109 (Blue), *B. japonicum* USDA6^T^ (Red), *B. diazoefficiens* USDA110 (green) and *B. diazoefficiens* USDA122 (purple). **(b)** Colony Forming Units per mL from a) of culture are plotted as a function of time. **(c)** Photograph of typical cell aggregates observed for the four isolates that spontaneously appear during the first 48hs accompanying the OD reduction (left). Microscopy observation of *B. japonicum* E109 aggregates using 1000X magnification (right). The photograph shows the cellular nature of aggregates. The black bar indicates 10 μm. **(d)** Generation times were calculated from several manual growth curves. The graph shows the box & whiskers plot minimum to maximum showing all the individual values. The central line indicates the median (*n* ≥ 6). Statistical significance was analyzed using Kruskal-Wallis non-parametric tests followed by Dunn’s multiple comparisons. *** means *p* < 0.001

We performed manual growth curves following OD_450nm_ over time on the earlier described temperate isolates of *Bradyrhizobium*: *Bj*6, *Bj*E109, *Bd*110, and *Bd*122. In the initial 20 to 24 h, we noticed that the cultures of the four strains display a decrease in OD_450nm_ (Fig. 1a).

The reduction in OD was quantified for all 4 strains and corresponded to a clearing in the growth media. To test if OD_450nm_ evolution accompanied population growth we plated bacteria along the growth curve. Throughout most of the experiment, colony forming unit (CFU) count followed the OD_450nm_ evolution (Fig. 1b). However, during the first 24 h, during the lag phase the CFU counts increased or remained stable. This phenomenon was not exclusive of the classic YEM medium; it also occurred in other media such as AG or in YEM with alternate carbon sources (data not shown). The OD_450nm_ reduction was accompanied with the appearance of aggregates (Fig. 1c). These aggregates were not inorganic precipitates of the medium components, as it can be normally observed, since under the microscope they clearly corresponded to *Bradyrhizobium* as shown by cell shape (Fig. 1c). In sum, *Bradyrhizobium* displays a distinctive lag phase in which cells do not die but aggregate, clarifying the medium. Hence, during this period CFU evolution does not correlate to OD.

### *B. Japonicum* tend to have a faster growth than *B. diazoefficiens*

We compared the growth rate of these four strains computing the generation time obtained from manual growth curves of OD over time in several experiments. Results are synthesized in Fig. 1d and in Table 1.

**Table 1:** Generation time of *Bradyrhizobium* strains cultivated in classic YEM medium

	Generation time (hours)				
Strain	Average	Median	SD	N	
*Bj*E109	15.7	15	2.745	11	
*Bj*6	9.4	9.3	1.631	5	
*Bd*110	17.8	17.3	4.054	8	
*Bd*122	18.8	19.9	3.532	5	

We observed that growth rate was not uniform in all the isolates. Notably, *Bj*6 displayed the lowest generation time of approximatively 9.4 ± 1.6 hs. *Bj*E109 showed a doubling time of 15.7 ± 2.7 hs. Meanwhile, with a generation time of 17.8 ± 4 hs. and 18.8 ± 3.5 hs., *Bd*110 and *Bd*122 respectively displayed the slowest growth among the tested strains. Statistical analysis showed that *Bj*6 was significantly faster that *Bd*110 and *Bd*122. Meanwhile, *Bj*E109 showed a growth rate that was not statistically different of either group although there is a biological trend in our experiments for this strain to grow slower than the former but faster than the *B. diazoefficiens* strains. Despite these strains displayed a wide variability between experiments, we observed that *B. japonicum* tends to be faster than *B. diazoefficiens*.

### Nutrient-dependent growth inhibition

YEM, the classical medium for *Bradyrhizobium* cultivation, is a minimal medium that allows robust but slow and variable growth of these microorganisms [37]. To facilitate its cultivation, we aimed at improving its composition. Two critical components of YEM are mannitol, as carbon source, and yeast extract (YE) as source of vitamins and micronutrients [38]. Also, gluconate (Glc) has been recommended to improve growth of slow-growing rhizobia. Previous studies showed that, in YEM, mannitol is in excess and does not condition Bradyrhizobial growth [37, 39]. They suggest also suggest that YE content influences Bradyrhizobial growth [38].

**Fig. 2:**
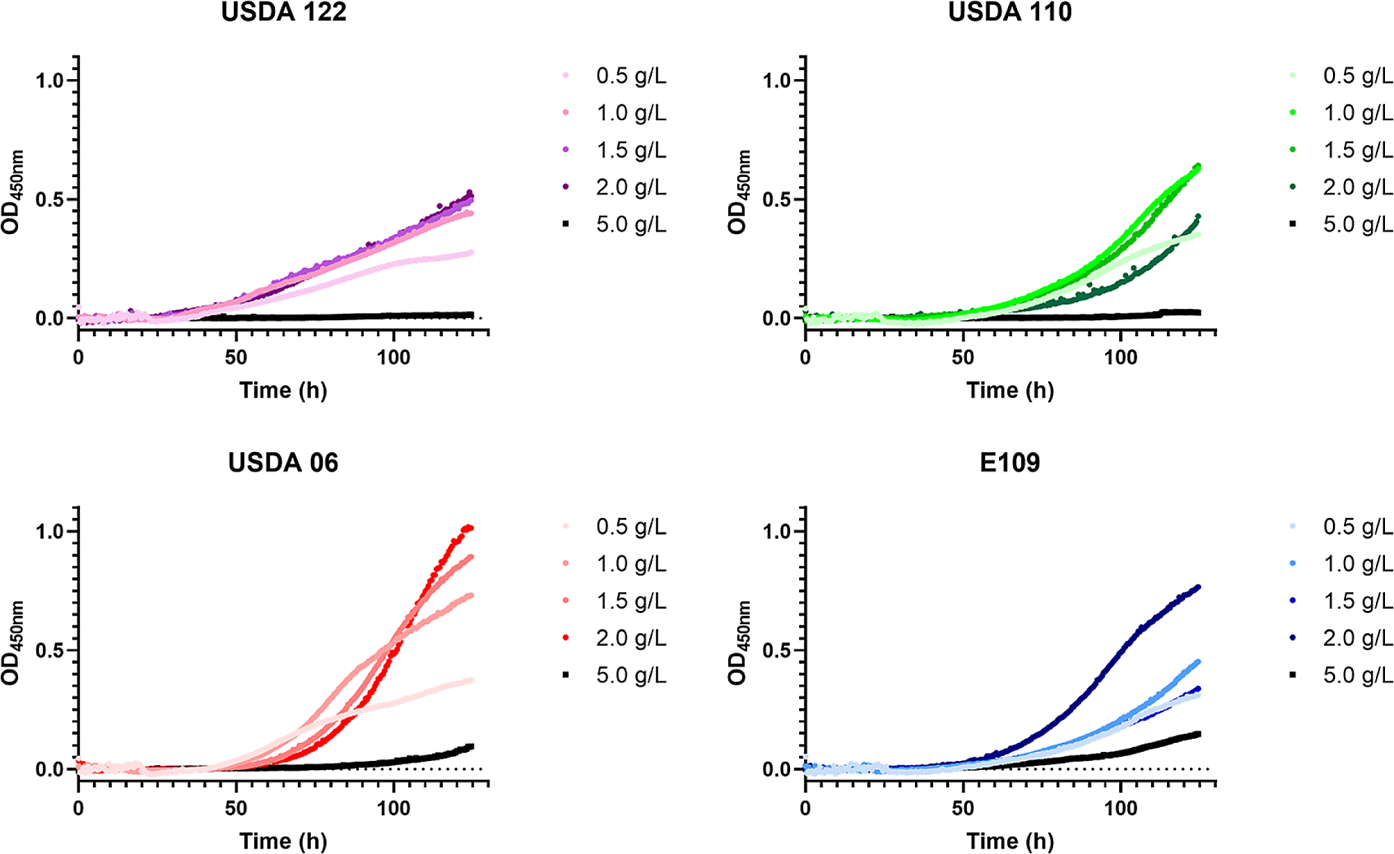
Increasing nutrient concentration inhibits*Bradyrhizobium* growth. Growth curves plotting OD_450nm_ as a function of time (in hours) of Bd1110 (shades of green) and, Bj109 (shades of blue) in manual growth curves. Strains were grown on increasing yeast extract concentration as indicated by darker colors. Upper panels show representative growth curves out of 3 performed that were used to calculate the GT values. The lower panels plot the mean value of generation times for both strains at the indicated YE concentration. Statistical significance was analyzed using One-way ANOVA and the Tukey test for multiple comparisons. Letters denote groups displaying statistically significant differences (P of at least < 0.05)

To test this, we assayed the growth of *Bradyrhizobium* in modified YEM medium increasing YE concentration. First, we tested *Bj*E109 and *Bd*110 using manual growth curves which allow optimal aeration, critical to obtain faster growth rates (Fig. 2). One would expect that increasing nutrient concentration would lead to higher growth until reaching the maximum growth capacity [1, 40, 41]. Growth curves were performed increasing YE content from its original concentration (0.5 gr/L) up to 5 gr/L (10 times more, but similar to amount used in the classical Lysogeny Broth). Notably, YE addition improved the growth of both *Bradyrhizobium* strains until reaching a maximum at 1.5 gr/L displaying a GT of 12.32 ± 0.57 hs. for *Bj*E109 and 11.32 ± 0.73 hs. for Bd110 (Fig. 2). After 2 gr/L, further addition of YE repressed growth (16.76 ± 0.6 gr/L for *Bj*E109) or it completely inhibited growth in the case of *Bd*110 at 5 gr/L (Fig. 2).

To generalize our observations to other isolates of the species, we extended our study to include *Bd*122 and *Bj*6 by performing automated growth curves increasing YE. For instance, the optimal growth of *Bd*110 and *Bd*122 occurred at 1.5 gr/L, but at 2gr/L, growth started to be inhibited, ultimately being arrested at 5 gr/L (Additional File 1: Figure S1). A similar situation was observed for *Bj*E109 and *Bj*6, except that, the maximum growth occurred at 1.5 and 2.0 gr/L, and that growth was not fully inhibited at 5 gr/L (Additional File 1: Figure S1). As a general trend, we observed that *Bradyrhizobium japonicum* tolerated higher nutrition concentration and developed higher growth rates than *Bradyrhizobium diazoefficiens*.

To test if this phenomenon was a peculiarity of YE or could be noticed with other nutrients we also tested growth under increasing concentrations of Glc. We tested concentrations ranging from 0.01 to 1% m/v. Overall, results were similar but to a lesser extent than with the addition of YE. Supplementing the media with extra Glc improved growth in all 4 strains, but a concentration of the compound beyond 0.1% m/v it inhibited *Bradyrhizobium* growth (Additional File 1: Fig. S2).

This set of experiments allowed us to obtain an improved YEM formulation using 1.5 gr/L of YE and 0.1% of Glc that optimized *Bradyrhizobium* growth using the optimal concentration of both medium components.

### Bradyrhizobia cell size does not correlate with cell age

**Fig. 3:**
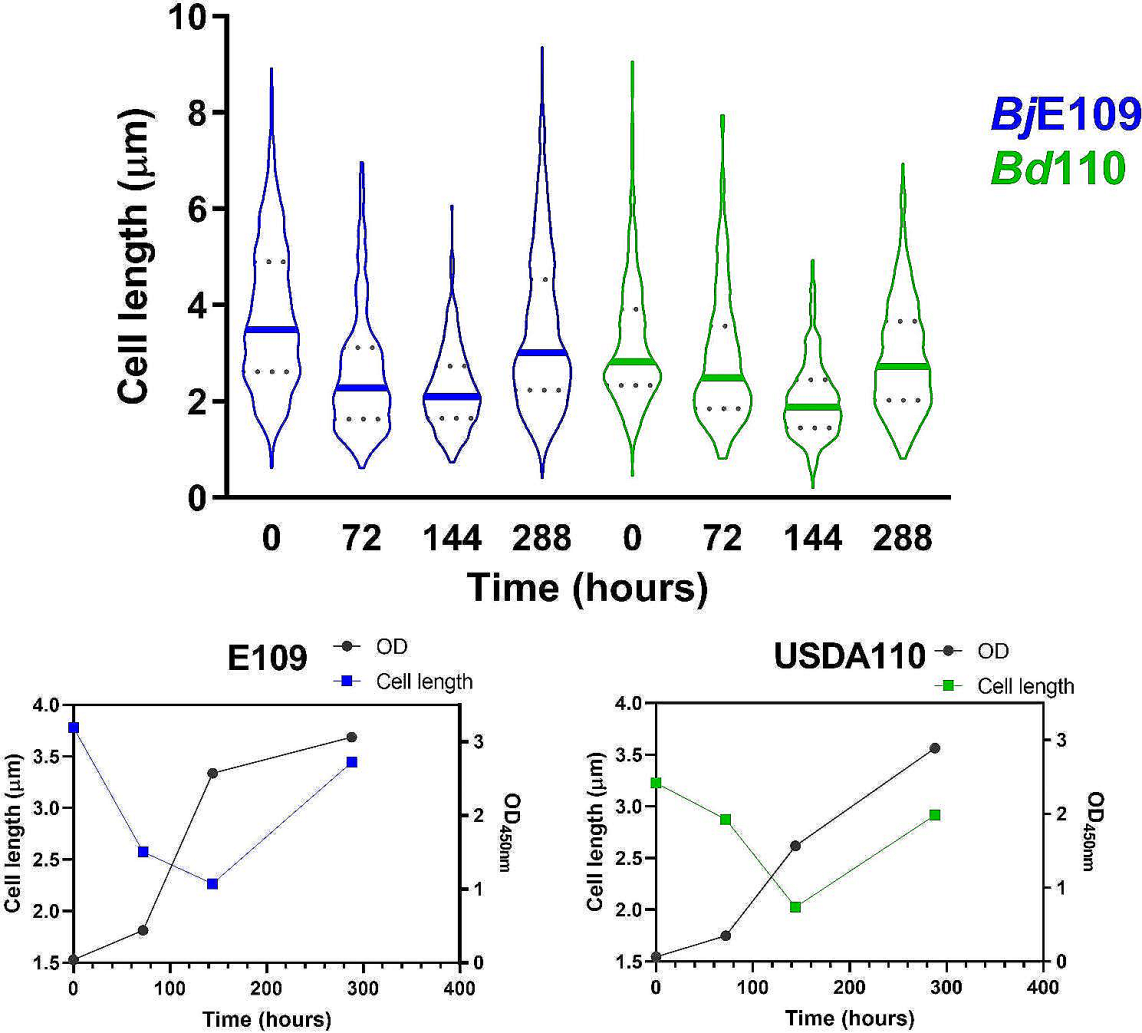
Cell size dynamics along the growth curve. *Bd*110 (green) and *Bj*E109 (blue) were grown on YEM medium. Samples were taken at different phases of the growth curve. Cells were photographed under the microscope and the OD_450nm_ of the culture was measured. (a) The distribution of individual cells lengths of *Bd*1110 (green, USDA110) and, *Bj*109 (Blue E109) was plotted along the experiment. The thick lines indicate the median for each timepoint. Dotted lines show the quartile range. Descriptive statistics can be found in Additional File 1, Table S2. (b) Each median of cell length (left axis) is plotted as a function of OD_450nm_ (right axis) is plotted as a function of time for each strain

Bacteria usually divide by binary fission which leads to two equally sized symmetric daughter cells. In most of the best-known models, size positively correlates with cell age [6]. Meanwhile, many α-Proteobacteria have been shown to present an asymmetric division with a new cell smaller than the mother cell [42]. To characterize cell division in *Bradyrhizobium*, we followed the cell size along 12 days (288 h) of growth curve by contrast microscopy for strains *Bd*110 and *Bj*E109 in YEM (Fig. 3a and Additional File 1: Table S2). Initially cell reduced their size between day 0 and day 3 (72 h) until day 6 (144 h). Subsequently, between day 6 and day 12 cell size increased again returning to initial length values. By comparing the obtained cell length to OD_450nm_ values, we can correlate cell size to the growth curve (Fig. 3b and c). Interestingly, bradyrhizobia reduced cell length upon entering exponential phase. Then cell size increases again during the transition between late exponential and stationary phase. This is opposite to what is observed to well-known models such as *Escherichia coli*. Meanwhile, *B. japonicum* E109 shows a larger cell size than *B. diazoefficiens* USDA110 during most of the experiment, particularly in exponential phase (Fig. 3a). This behavior was not specific to the medium or strain, since the same trend was observed when the experiment was performed in AG medium using *Bd*110, *Bd*122, *Bj*6, and *Bj*E109 (Additional File 1: Figure S3).

### Bradyrhizobia isolates display large fitness differences that correlate with growth rate and cell size

Growth rate is a good estimator of cells fitness although it must be taken with caution [43]. However, it does not take into account advantages occurring beyond balanced growth (i.e. outside the exponential growth phase). Additionally, we observed varying growth rates and cell sizes among each of the *Bradyrhizobium* isolates. In the case of *Bj*E109, we observed a repeated trend, though not statistically significant, of a faster growth than Bd*110* and *Bd*122.

**Fig. 4:**
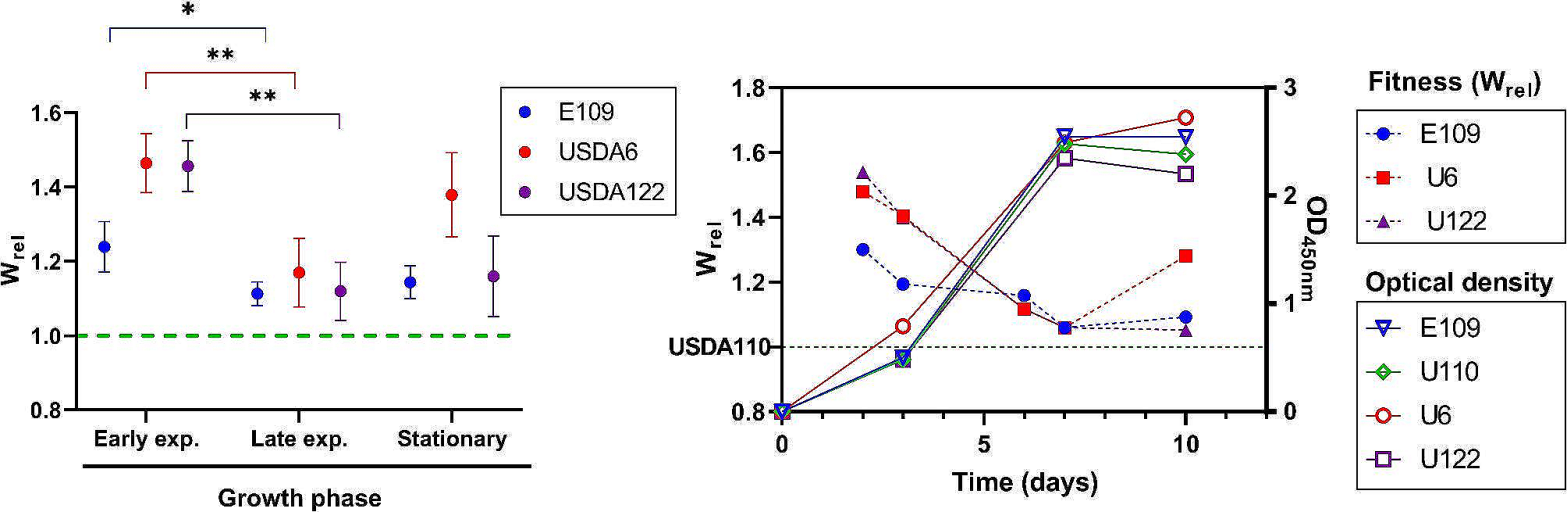
B. *japonicum* tend to display a fitness advantage over B. *diazoefficiens* during lag to early-exponential phase. Pairwise competition experiments between *Bd*110::*gfp* + and *Bd*122 (purple), *Bd*110 (green), *Bj*6 (red) and, *Bj*109 (blue) was performed as described in material and methods. For each time point OD_450nm_ was determined. **(a)** Relative fitness at early (3 days), late exponential (7 days) and stationary phase (10 days) of growth in co-culture. The Bd110 against the same strain expressing *gfp* was taken as reference for relativizing fitness (green dotted line). Points represent mean with SEM (*n* = 4). Statistical differences were computed using Two-way ANOVA and Holm-Sidak for multiple comparisons. * and ** mean *p* < 0.05 and *p* < 0.01 respectively. **(b)** A representative dataset where W_rel_ (left axis, filled dots, dotted connecting lines) and OD_450nm_ (right axis, solid connecting lines, empty dots) at each time point were plotted as a function of elapsed time since the beginning of the experiment

To disentangle both questions simultaneously, we measured the fitness of *Bj*6, *Bj*E109, *Bd*110, and *Bd*122. In this aim, we assessed competitiveness in pair-wise competitions. We used a GFP-tagged *Bd*110 (Additional File 1: Table S1) to co-culture it in equal amounts with each one of the aforementioned *Bradyrhizobium* strains. Then, we grew the cells for two weeks and, using fluorescence microscopy, we monitored deviations from a 1:1 ratio during this period until arriving to stationary phase (OD_450nm_ ≈ 2.5). Next, we calculated the absolute fitness (W) from these deviations and then we relativized to the competition *Bd*110 against *Bd*110::*gfp +* to obtain a relative competitivity index (W_rel_, see methods). As a general trend, we observed that *Bd*110 is outcompeted by the rest of the isolates in all the growth phases (Fig. 4a). In all data points the W_rel_ was > 1. Also, *Bj*6 is significantly fitter that the rest of the strains, displaying the highest competitivity index at the end of the experiment (Fig. 4a and Additional File 1: Figure S4 and Table S3). While there are not statistically significant differences between *Bj*E109 and Bd122, the former tends to be more competitive than the latter, showing higher W_rel_ in 2 out of 3 experiments (Additional File 1: Figure S4 and Table S3).

The slow growth of *Bradyrhizobium* allows to observe the moment of the growth curve where the strains display highest fitness differences. Interestingly, the highest differences are observed at the beginning of early exponential phase, suggesting that the competitiveness differences occur during lag phase (Fig. 4b) and then diminish as the growth curve progresses.

### Bradyrhizobia present division asymmetry and an unusual cell cycle

The observation that bradyrhizobia cell size does not correlate to cell age (Fig. 3) led to pursue a better characterization of its entire cell cycle. In this aim, we performed time lapse microscopy of *Bj* E109 and *Bd*110. For this, we imaged cells on agar pads, taking photographs every 30 min for 5 to 6 days. Under our experimental conditions, we noticed that cell duplication is not a continuous nor homogeneous process; it does not occur uniformly across all cells in a similar way. Some cells replicate more actively than the rest of the population. Cells under the microscope showed periods of replicative burst and periods of relative quiescence. These periods were not similar for all cells observed, and we noted that bacteria tend to cluster closely and approach each other. To illustrate this we present a representative video of the many movies performed. (Video S1). Meanwhile, the division of the cells clearly differs from binary fission [44–46] since we observed an heterogenous behavior and a large asymmetry with long mother cells producing somewhat smaller daughter cells.

**Fig. 5:**
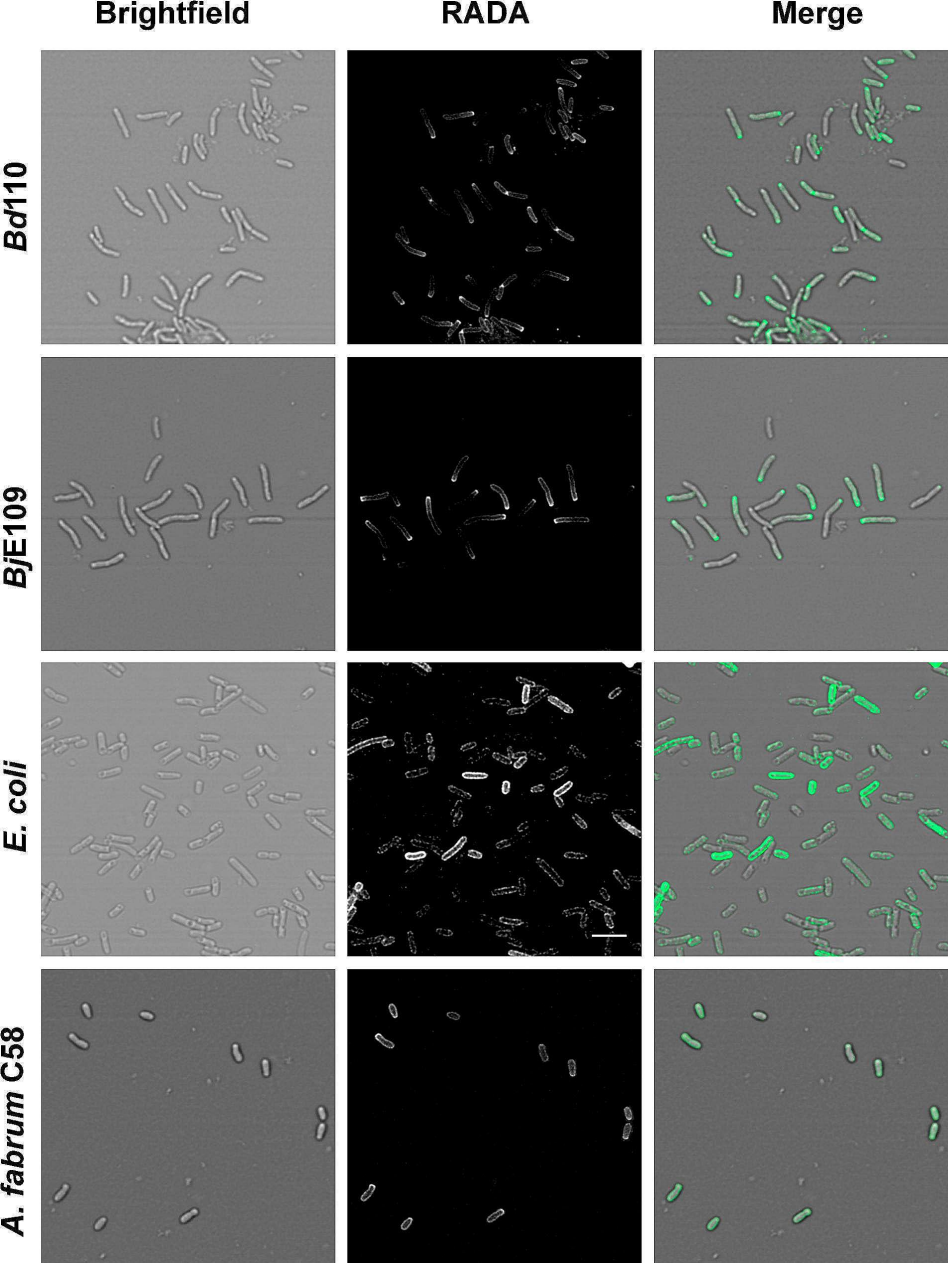
*Bradyrhizobium* present an extreme asymmetry. Cells were stained with sBADA and photographed using CLSM as indicates un material and methods. *A. fabrum* C58 an asymmetric α-Proteobacterium was used as a control of an asymmetric cell and *E.coli* as a regular bacterium dividing by binary fission. The white bar at the central panel corresponds to 5 μm for all panels

To determine the differences in cell cycle and division in more detail, we cultured *Bd*110 and *Bj*109 in the presence of the fluorescent D-amino acid sBADA [47]. D-amino acids only incorporate into the cell wall of actively replicating cells; therefore, sBADA specifically labels actively replicating sites of cells [47]. For comparative purposes we employed *Escherichia coli* and *Agrobacterium fabrum* C58 (formerly known as *A. tumefaciens*). *E. coli* is a well-known Gram-negative bacteria that divides by binary fission. Meanwhile, *A. fabrum*, like other α-Proteobacteria, displays a polar growth that has been well characterized by other laboratories [42]. Figure 5 shows a confocal laser scanning microscopy (CLSM) images of cells in exponential phase stained with sBADA. *E. coli* shows a homogeneous stain along the cell. In *A. fabrum* C58 the fluorescence signal is present throughout the entire cell. However, as expected due to its asymmetrical growth, an increase in the sBADA signal can be observed at the growing (new) pole. *Bradyrhizobium* isolates displayed a particular signal pattern. Cells are longer and an increasing gradient of fluorescence signal along the longitudinal axis of the cell is observed from old to the new pole. The maximum sBADA signal is very strong at the new pole. Therefore, we find that the stain differs between *Agrobacterium* and *Bradyrhizobium* with a more pronounced asymmetry in the latter. This is better observed when cells are incubated constantly with the fluorescent D-amino acid (Additional File 1: Figure S5).

**Fig. 6:**
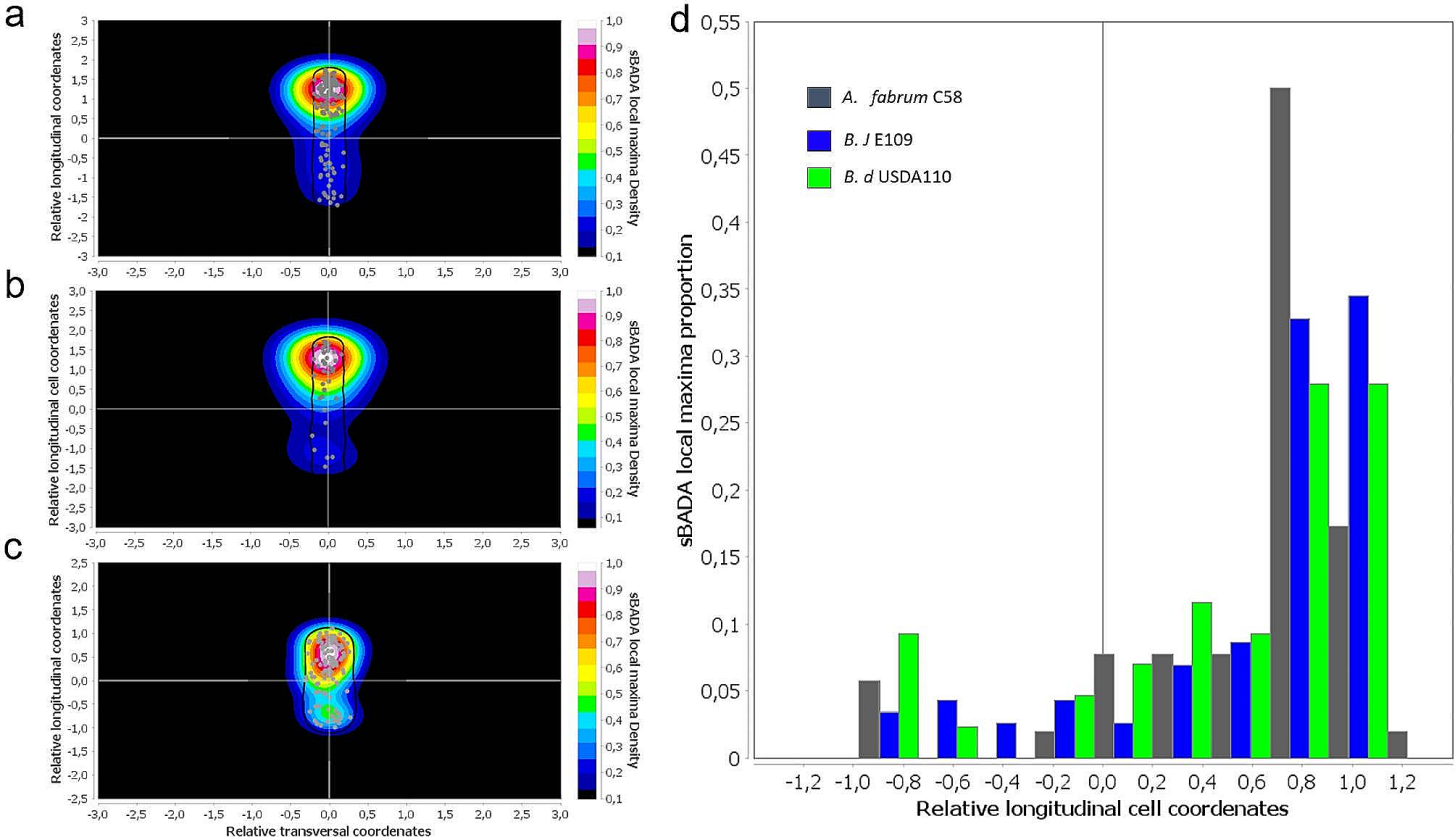
*Bradyrhizobium* displays stronger growth asymmetry than *Agrobacterium fabrum* C58. Using FIJI plugin MicrobeJ [48], we built an sBADA local maxima density heatmap in *B. japonicum* E109 **(a)** (*n* = 162), *B. diazoefficiens* USDA110 **(b)** (*n* = 86) and *(A) fabrum* C58 **(c)** (*n* = 90) cells. Grey dots represent events of local maxima detected within the cell. **(d)** Proportion histogram of local maxima events shown in **(a)**, **(b)** and **(c)**, along *(B) japonicum* E109, *B. diazoefficiens* USDA110 and *A. fabrum* C58 cells. Cell poles 1 and − 1 were defined by intensity of sBADA channel, being cell pole 1 the one with higher intensity. The vertical grey line denotes the cell center. We used the same threshold settings of intensity and Z-score for sBADA maxima detection in every experiment

To better assess these differences, we analyzed the images taken and quantified the sBADA signal using MicrobeJ [48]. This allowed us to quantify differences in the signal between bradyrhizobia and *Agrobacterium fabrum* C58. The sBADA signal maxima are located next to the new pole both in the case of Bj109 (Fig. 6a) and Bd110 (Fig. 6b). *A. fabrum* C58 displays an asymmetric signal that is more disperse and not as close to the new pole (Fig. 6c). When the three strains are compared by normalizing their length (Fig. 6d), it becomes clear thar signal maxima of Bj109 and Bd110 occur around the new pole, denoted by 1, whereas in the case of *A. fabrum* it is found at 0.6. This shows that the *Bradyrhizobium* strains display a stronger asymmetry than *Agrobacterium*.

This strong asymmetric cell growth pattern is similar in both bradyrhizobia studied (Figs. 5 and 6) strongly suggesting that this is a common behavior of the genus.

## Discussion

Bacterial growth and cell cycle has been widely studied in specific models such as *E. coli*, *Salmonella enterica, Bacillus subtilis* and *Caulobacter vibrioides* [5, 49–51]. Information for other bacterial species has also been collected but their study remains much more limited [52, 53]. In particular, studies on *Bradyrhizobium* physiology, particularly those quantitative, remain scarce and mostly limited to *Bd*110 [21, 53]. Also, reports on *Bradyrhizobium* physiological parameters, particularly generation time, remain highly variable across the literature.

Here, we conducted a comparative study of the general physiology of 4 strains that display the representative genomic profiles [29] and thrive in temperate environments. We devoted particular attention to *Bd*110 since is the best studied strain and to *Bj*E109 due to its great agronomical importance [17, 28, 54]. While it is tempting to generalize our conclusions to the whole genus, our study is limited to two closely related species, *B. japonicum* and *B. diazoefficiens*, belonging to the same superclade (japonicum, superclade I). To achieve a more comprehensive understanding, it would be necessary to extend our study to include other groups of the genus, such as *Bradyrhizobium elkanii* (elkanii, superclade II), *Bradyrhizobium oligotrophicum* (the photosynthetic, superclade III), and *Bradyrhizobium lablabi* (the extra-slow growers, superclade IV) [14]. However, this is beyond of the scope of our current work, which primarily focuses on temperate strains commonly employed as soybean inoculants.

We employed 4 different approaches to quantify the general physiology of these species. Overall, a consistent trend emerged, with *B. japonicum* displaying a faster growth than *B. diazoefficiens* isolates: they exhibited a lower doubling time both in manual and in automated growth curves (Figs. 1 and 2; Table 1); in line with bacterial “growth laws” that correlate growth to cell size [5], they displayed larger cell size in exponential phase (Fig. 3 an d Additional File 1: Figure S3); they tolerated higher nutrient loads (Fig. 2 and Additional File 1: Figures S1 and S2); and, outperformed in pairwise competition (Fig. 4 and Additional File 1: Table S2). While each individual approach did not display drastic differences a consistent trend was observed across all experiments. Genomic studies have previously linked growth rate to the ribosomal RNA operon (*rrn*) ploidy [7]. Indeed, experimental work has shown this in several bacterial models, although some controversy remains regarding whether *rrn* ploidy affects doubling time or lag phase duration [55–58]. In either case, *B. japonicum* displays 2 *rrn* while *B. diazoefficiens* harbors a single *rrn* copy (Additional File 1: Figure S6). This genomic difference aligns with the observed faster growth of *B. japonicum* (Figs. 1 and 2), cell size in exponential phase (Fig. 3) and superior competitiveness (Fig. 4). In this latter experiment, we observed that the other *Bradyrhizobium* strains outcompete *Bd*USDA110. The slow growth of *Bradyrhizobium* allowed us to assess fitness differences at different stages of the growth curve. We noticed that the most fit strains gained an advantage at the beginning of the experiment with the majority of fitness gain for strains *Bd*122, *Bj*E109 and *Bj*6 occurring during lag phase (Fig. 4b). This is consistent with previous studies showing that *rrn* ploidy impacts lag phase rather than doubling time [57].

*B. japonicum* strains displayed more tolerance to high concentration of nutrients. In this regard, we found a peculiarity in bradyrhizobia. It is normally assumed that higher nutrient concentrations lead to faster growth, as reflected in Monod curves [1]. Unexpectedly, we observed that increasing concentrations of gluconate or yeast extract above the optimal level reduce the growth rate of *Bradyrhizobium* (Fig. 2 and Additional File 1: Figure S1). Such behavior could explained by the ecological role of bradyrhizobia if we think on them as exclusive oligotrophic bacteria that competes with copiotroph for the same resources [41, 59]. *Bradyrhizobium* would be more efficient at low concentrations that their copiotroph counterparts at low nutrient concentration. However, higher nutrient concentration might be toxic for bradyrhizobia due to exacerbated transporters that would allow the entry of certain nutrients beyond the levels tolerable by cell metabolism.

Another anomaly in *Bradyrhizobium* growth was the formation of cellular aggregates at the beginning of the exponential phase (Fig. 1c and d). This phenotype deserves further exploration beyond the present study. Cell aggregation could contribute to growth. Indeed, it is observed during cell plating that denser bacterial suspensions take 3–4 days to develop while in most diluted bradyrhizobial suspensions can take 1–2 weeks to develop colonies in the plate. Therefore, bradyrhizobial replication could be density-dependent. It could be driven either by quorum sensing (QS) and/or cell-cell contact. The N-acyl homoserine lactones, the QS signaling molecules, have been shown to alter swimming, aggregation or biofilm formation but not growth of *Bradyrhizobium* [60, 61]. Meanwhile, a mild cell cycle acceleration due to cell contact with surfaces has been observed in other α-Protobacteria [62].

Overall, we have better characterized *Bradyrhizobium* growth and successfully described its particular cell cycle with an asymmetric division that is more pronounced than in other α-Proteobacteria such as *Agrobacterium fabrum* C58 (Figures, 5 and 6). To fully characterize *Bradyrhizobium* cell cycle, it remains to mark other cell components such as DNA and to describe chromosome choreography and spatial structure (63–65).

Our experiments suggest that *rrn* ploidy may shape the physiology of brayrhizobia. Using 4 different approaches (cell size, growth rate, nutrient concentration tolerance, and pairwise) we observe the same trend: *B. japonicum* (2 *rrn*) displays faster growth than *B. diazoefficiens* (1 *rrn*). While the differences are not always statistically significant, we find it suggestive that all these different approaches provide similar results. We plan to investigate this phenomenon in further works by deleting or adding *rrn* copies within the same genetic background. We do not mean that *rrn* ploidy is the main factor driving cell physiology. For instance, *Bj*6 displayed the fastest growth among the strains in our working conditions, although it displays the same *rrn* structure than *Bj*E109. Meanwhile, *rrn*, among other factors such as codon usage, tRNA ploidy, and genomic location of transcription and translation genes strongly condition cell physiology [3, 4, 35, 55, 66] and evolution [67]. There very are few studies linking the chromosome structure and gene order to cell physiology. Most of the insight comes from well-known model organisms such as *E. coli* and *B. subtilis*. The present work initiates the physiological characterization of an extremely-slow growing culturable organism representing a first step towards exploring this subject in such economically relevant microorganism. Indeed, finding ways to engineer these microorganisms to grow faster could have great biotechnological potential by improving inoculant production.

### Electronic supplementary material

Below is the link to the electronic supplementary material.

Supplementary Material 1

Supplementary Material 2

Supplementary Material 3

## Data Availability

All data generated and analysed during this study are included in this published article and its supplementary information files.
