# Supplementary material for: The distinct cell physiology of Bradyrhizobium at the population and cellular level
Source: BMC Microbiol. 2024 Apr 20;24:129. doi: 10.1186/s12866-024-03272-x (PMC11031950; doi:10.1186/s12866-024-03272-x)
Supplement: Supplementary file 2 — Supplementary Material 2 [file 12866_2024_3272_MOESM2_ESM.docx]

**Video legends:**

**Video S1: A representative video microscopy of *Bradyrhizobium japonicum* E109.** An exponentially growing culture was distributed in an AG-agar layer and kept at 28°C. Images were recorded every 30 minutes as indicated in material and methods. Time in hours is indicated in the upper left corner. The white bar in the lower right part represents 5 µm. Green arrows indicate actively replicating microcolonies. Yellow arrows indicate microcolonies that only perform a few replication rounds. Red arrows indicate non-dividing cells.
