# Supplementary material for: The distinct cell physiology of Bradyrhizobium at the population and cellular level
Source: BMC Microbiol. 2024 Apr 20;24:129. doi: 10.1186/s12866-024-03272-x (PMC11031950; doi:10.1186/s12866-024-03272-x)
Supplement: Supplementary file 3 — Supplementary Material 3 [file 12866_2024_3272_MOESM3_ESM.docx]

**Supplementary Material for:**

**“The distinct cell physiology of *Bradyrhizobium*** **at the population and cellular level.”**

Ian F. Medici^1^*, Leila Bartrolí^1^*, Fabiana R. Fulgenzi^1^, Francisco Guaimas^1^, Charo Luciana Molina^1^, Ignacio E. Sánchez^2^ Diego J. Comerci^1^, Elías Mongiardini^3^ & Alfonso Soler-Bistué^1^

^1^ Instituto de Investigaciones Biotecnológicas “Dr. Rodolfo A. Ugalde”, IIB-IIBIO, Universidad Nacional de San Martín- Consejo Nacional de Investigaciones Científicas y Técnicas (CONICET), San Martín, Buenos Aires, Argentina.

^2^ Laboratorio de Fisiología de Proteínas, Facultad de Ciencias Exactas y Naturales, CONICET, Instituto de Química Biológica, Facultad de Ciencias Exactas y Naturales (IQUIBICEN), Universidad de Buenos Aires, Buenos Aires, Argentina.

^3^Instituto de Biotecnología y Biología Molecular (IBBM), Facultad de Ciencias Exactas, UNLP y CCT-La Plata-CONICET, La Plata, Argentina


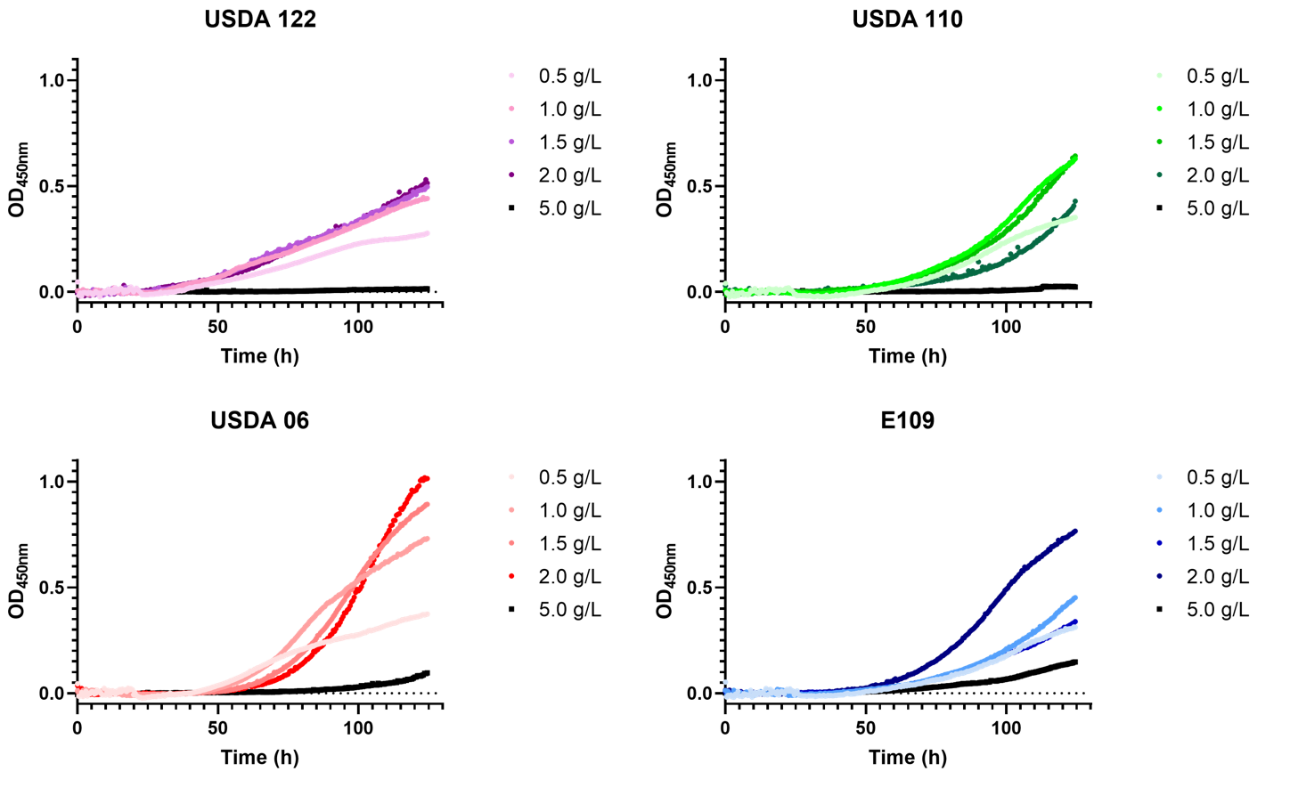


**Figure S1: Increasing YE concentration inhibits *Bradyrhizobium* growth**. Growth curves plotting OD_450nm_ as a function of time (in hours) of *Bd*122 (shades of purple), *Bd*1110 (USDA 110 shades of green), *Bj*6 (USDA 06, shades of red) and, *Bj*109 (E109, shades of blue). Strains were grown on increasing yeast extract concentration as indicated by darker colors. The graph shows a representative experiment out of 3 performed.

**
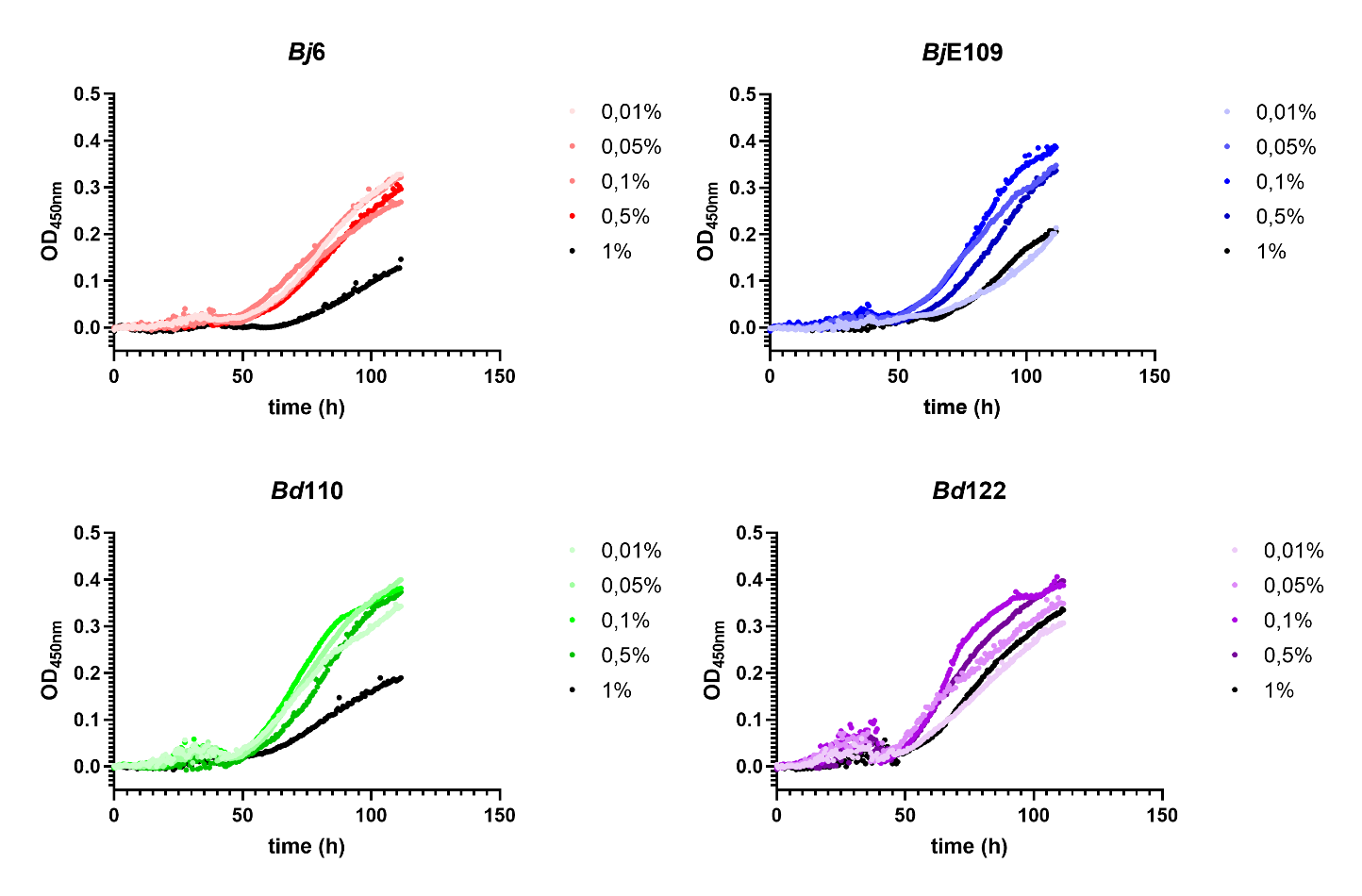
**

**Figure S2: Increasing gluconate concentration inhibits *Bradyrhizobium* growth**. Growth curves plotting OD_450nm_ as a function of time (in hours) of *Bd*122 (purple nuances), *Bd*1110 (shades of green), *Bj*6 (shades of red) and, *Bj*109 (shades of blue). Strains were grown on increasing sodium gluconate concentration as indicated by darker colors. The graph shows a representative experiment of 2 performed in triplicates.


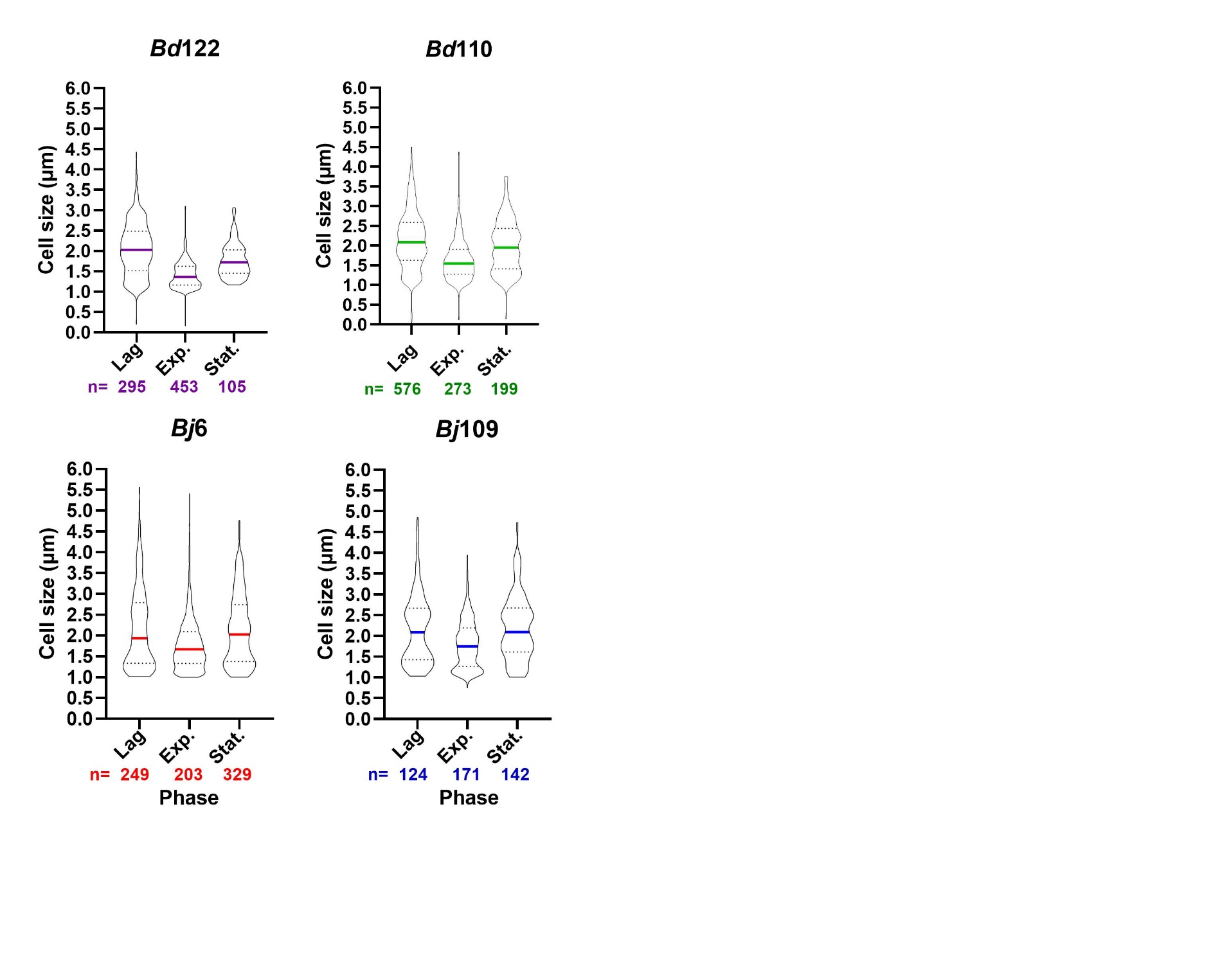


**Figure S3** **Bradyrhizobial cell size at different stages of culture growth:** Cells of *Bd*122 (purple), *Bd*110 (green), *Bj*6 (red) and, *Bj*109 (blue) were grown in AG medium and registered at the indicated growth phases (lag, exponential, and stationary phase). The median of cell length (μm) is plotted for each growth stage for each one of the strains. Violin plots indicate the median of at least 150 cells. Dotted lines indicate interquartile range.

**Figure S4: Individual W values of competition experiments.** *Bj*109 (blue), *Bd*110 (green), *Bj*6 (red) and, *Bd*122 (purple) were subjected to pairwise competition against *Bd*110::*gfp*^+^ strain in YEM medium for 10 to 12 days. The competition index (W) was calculated as indicated in the Material & Methods section. The graph displays the individual W values obtained. Statistical differences were computed using Kruskal-Wallis and Dunn’s Test for multiple comparisons. * indicates p<0.05.


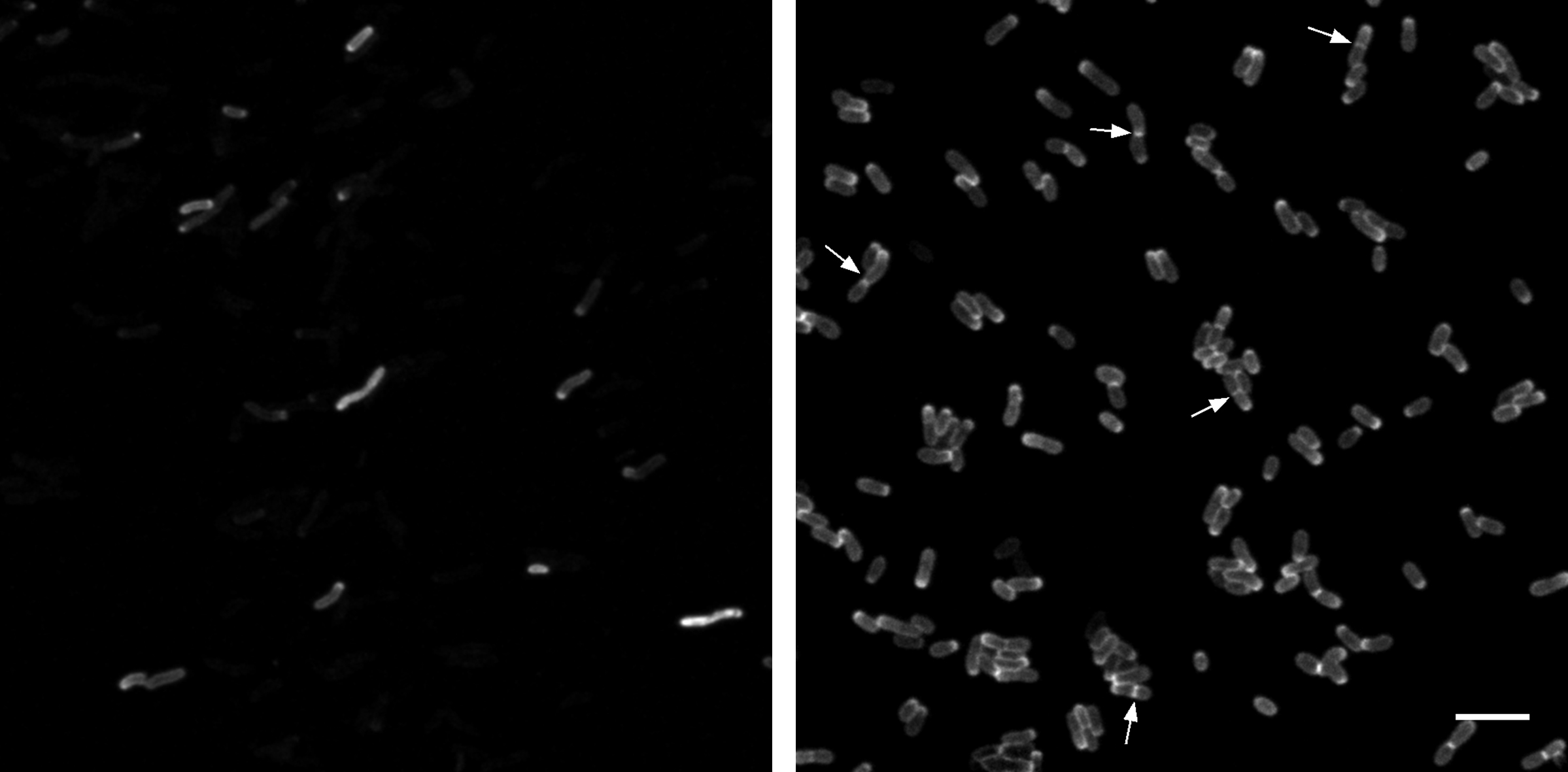


**Figure S5: *Bradyrhizbium* presents a more pronounced asymmetry than *Agrobacterium fabrum*.**  *B. japonicum (*Left panel*)*  and *A. fabrum* (Right panel) cells were cultured to exponential phase as in previous experiments except that instead of a pulse during the 10% of doubling time, we constantly provided Orange-red TAMRA-based fluorescent D-amino acid (RADA) in the culture media. The cells were washed and visualized using CLSM. The arrows indicate some constricted septa identified in *Agrobacterium*. The white line in the right panel indicates 5 µm.

### Figure S6: The *rrn* structure of Bradyrhizobia under study. The chromosomes of *Bd*122, *Bd*110, *Bj*6 and, *Bj*109 chromosomes in scale are displayed as circles. The numbering starts at the *oriC*. Them the inner circles show tRNAs and the rRNA operons (red triangles). The inner circle shows the GC sckew. This graph was created using Genovi (1)

**
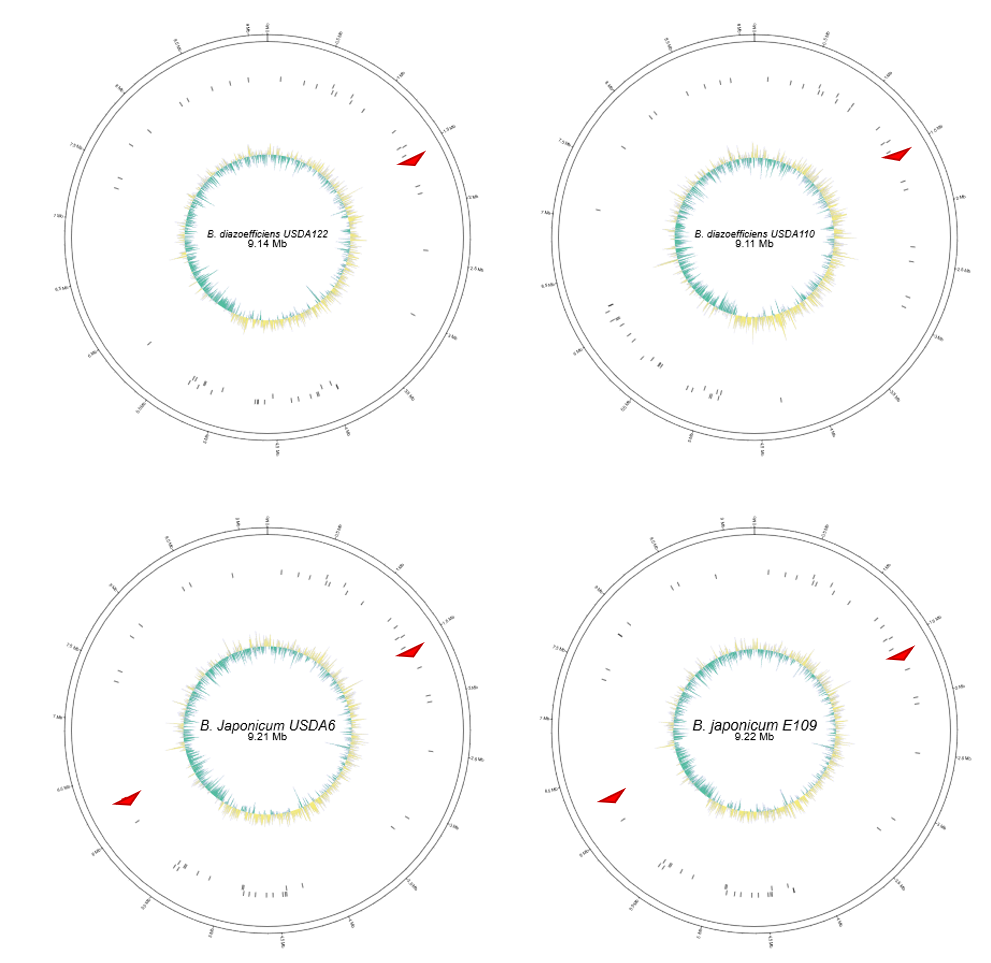
**

**Table S1:** Bacterial strains used in this study.

| **Strain** | **Relevant characteristics** | **Source** |
| --- | --- | --- |
| *B. diazoefficiens* USDA 110 | *Bradyrhizobium diazoefficiens* wild-type | Lodeiro Laboratory |
| *B. diazoefficiens* USDA 122 | *B. diazoefficiens* USDA 122 wild type | Lodeiro Laboratory |
| *B. japonicum* E109 | *B. japonicum* E109 wild type | Fabricio Cassán Laboratory |
| B. japonicum USDA6^T^ | *B. japonicum* USDA6^T^ wild type | Lodeiro Laboratory |
| *B. diazoefficiens* USDA 110 *gfp*^+^ | GFP^+^ | Lodeiro Laboratory. |
| *E. coli* DH5*α* | Model for bacterial growth by binary fission | Comcerci Laboratory. |
| *Agrobacterium tumefaciens* C58 | Model for Assymmetric division | Comerci Laboratory |

**Table S2 Cell size dynamics along the growth curve.** This table summarizes the medians of cell length and the number of cells measured as indicated in the Figure 3 of the main text.

|  | **Day 0** | | **Day 3** | | **Day 6** | | **Day 12** | |
| --- | --- | --- | --- | --- | --- | --- | --- | --- |
|  | **BjE109** | **Bd110** | **BjE109** | **Bd110** | **BjE109** | **Bd110** | **BjE109** | **Bd110** |
| **cell length (μm)** | 3.48 | 2.81 | 2.28 | 2.49 | 2.09 | 1.88 | 3.01 | 2.72 |
| **n** | 313 | 305 | 309 | 323 | 300 | 327 | 348 | 369 |

**Table S3, Competition experiment between different *Bradyrhizobium* strains.** The W_rel_ at the experiment endpoint is shown for each competing strain in descending order of competitiveness. Columns I to III represent independent experiments.

|  | W**_rel_** | | |
| --- | --- | --- | --- |
| Strain/Experiment # | I | II | III |
| 1)*Bj*USDA6 | 1,72 | 1,28 | 1,18 |
| 2)*Bj*E109 | 1,27 | 1,09 | 1,12 |
| 3)*Bd*USDA122 | 1,48 | 1,05 | 1,05 |
| 4)*Bd*USDA110 (reference) | 1 | 1 | 1 |

**Additional citations:**

1- Cumsille A, Durán RE, Rodríguez-Delherbe A, Saona-Urmeneta V, Cámara B, Seeger M, et al. “GenoVi, an open-source automated circular genome visualizer for bacteria and archaea.” PLoS Comput Biol. 2023;19:e1010998.
